# Inactivation and replantation of the knee joint: an infeasible surgical method

**DOI:** 10.1186/s12957-024-03311-x

**Published:** 2024-01-25

**Authors:** Zhichao Tian, Yang Yang, Peng Zhang, Xin Wang, Weitao Yao

**Affiliations:** 1 Department of Sarcoma, the Affiliated Cancer Hospital of Zhengzhou University & Henan Cancer Hospital, Zhengzhou, 450008 Henan Province China; 2 Modern Educational Technology Center, Henan University of Economics and Law, Zhengzhou, 450046 Henan Province China

**Keywords:** Inactivated, Autograft, Replantation, Osteosarcoma, Knee joint

## Abstract

**Background:**

The inactivation and replantation of autologous tumor bones are important surgical methods for limb salvage in patients with malignancies. Currently, there are few reports on the inactivation and replantation of the knee joint. In this study, we aimed to evaluate the feasibility of our surgical approach.

**Methods:**

This is a retrospective case series study. We retrospectively collected the clinical data of patients with sarcoma treated with knee joint inactivation and replantation and analyzed the efficacy of this surgical method. The bone healing and complications in these patients after inactivated autograft replantation were assessed.

**Results:**

This study included 16 patients. Fifteen patients had osteosarcoma, and one had Ewing's sarcoma. The average length of the inactivated bone is 20.2 cm (range 13.5–25.3 cm). All the patients underwent internal plate fixation. The average follow-up duration was 30 months (range 8–60 months). Before the data deadline of this study, eight (50%) patients were still alive, and eight (50%) died of sarcoma metastasis. Eight (50%) patients achieved bone healing at the diaphysis site of the inactivated tumor bone, with an average bone healing time of 21.9 months (range, 12–36 months). Five (31%) patients died due to metastases and did not achieve bone healing. Two (12.5%) patients did not achieve bone healing because of infection, and one (6.3%) patient underwent amputation due to tumor recurrence. Ten (62.5%) patients experienced fractures around the joint ends of the inactivated replanted bone, and eight of these ten patients were combined with joint dislocation.

**Conclusion:**

The incidence of joint deformities after the knee-joint inactivation and replantation is extremely high and is not recommended for use.

## Background

Although the incidence rate is low, tens of thousands of patients are newly diagnosed with malignant tumors involving the knee joint annually [1–3]. Many of these patients require complete resection of the tumor bone, repair of bone defects, and reconstruction of limb function [4–6]. Artificial prosthesis replacement is the main reconstruction method used after bone and joint resections [2, 7, 8]. However, in some patients, such as underage patients and those with excessively long tumor segments, the limitations of prosthesis replacement surgery are obvious (such as wear and loosening) [2, 7–9]. Among these patients, inactivation and replantation of the tumor bone are feasible options [5, 10, 11].

In clinical practice, cases in which the tumor bone cortex remains relatively intact and limb salvage is required can be considered for treatment with tumor bone inactivation and replantation. The basic principle of tumor bone inactivation and replantation is to use various physical methods to inactivate the tumor bone and then implant it in situ into the body. There are various methods for tumor bone inactivation, each with its own advantages and disadvantages [12–15]. Some studies suggest that frozen inactivation and cartilage replantation are feasible [16]. However, there are currently few reports on bone and joint inactivation surgery involving the articular surface; therefore, it is not clear whether inactivated replantation is feasible for the treatment of patients with malignant bone tumors involving the articular surface. As a tertiary referral center, we have used ethanol, irradiation, and frozen inactivation to treat many patients for over a decade, with some patients undergoing knee joint inactivation and replantation. In this study, we retrospectively collected and analyzed the clinical data of these patients to determine the feasibility of this surgical approach.

## Methods

### Patients

We retrospectively collected clinical data from patients who met the inclusion criteria and received treatment between January 2010 and May 2021. Only patient data that meets the following inclusion criteria will be collected: (1) received alcohol-, irradiation-, or frozen-inactivated tumor bone replantation surgery; (2) the inactivated replanted bon including the cartilage surface of the knee joint; and (3) complete follow-up data. Follow-up data for this study ended on August 30, 2023.

### Treatment protocol

All pathological diagnoses were confirmed via biopsy. All patients underwent pre- and post- operative chemotherapy.

The resection length was determined using preoperative magnetic resonance imaging. The tumor and surrounding normal tissues were removed as a whole, with a minimum edge of 2 cm. Any attached soft tissue or gross tumor was removed from the excised bone, and bone canals were scraped.

In patients who underwent frozen-inactivated tumor bone replantation, the tumor bone was frozen and inactivated using liquid nitrogen. Briefly, the bone was soaked in liquid nitrogen for 30 min and thawed at room temperature (24 °C) for 30 min. In patients who underwent irradiation-inactivated tumor-bone replantation, the tumor bone was fully and evenly wrapped in sterile saline gauze, making it a relatively uniformly dense specimen. The specimen was then sent to the radiotherapy center for irradiation inactivation. The tumor bone was subjected to isocenter-penetrating irradiation using an X-ray electron linear accelerator at a dose of 60 Gy, with an average irradiation time of 30 min and a dose rate of 2.0 Gy/min. In patients who underwent alcohol-inactivated tumor bone replantation, the tumor bone was prefixed with an appropriately sized plate. The tumor bone and plate composite were then immersed in alcohol for 40 min for inactivation. The inactivated tumor bone was implanted in situ into the bone defect and fixed with a plate.

The patients were encouraged to immediately begin a moderate range of exercises (those that were painless) postoperatively. Partial weight bearing was allowed 1 month after surgery. Radiological evidence of bone connection at the osteotomy site included blurred osteotomy lines or sufficiently bridging calli at the host-graft junction. Only when strong bone bonding is accomplished can full weight-bearing be attained.

### Collection and evaluation of clinical data

The baseline characteristics of all patients enrolled in this study were reviewed. Bone healing was evaluated using radiography every 3 months until the autograft healed. Follow-up was performed using computer tomography scan and ultrasonic examination every 3 months in the first year and then every 6 months to check for recurrence and metastasis. In this study, we assessed bone healing and complications in these patients after inactivated autograft replantation. Functional status was assessed using the Musculoskeletal Tumor Society (MSTS) scale, which is based on six parameters (pain, functional activity, emotional acceptance, use of external support, walking ability, and gait) [17].

Statistical analyses were performed using Statistical Package for the Social Sciences (SPSS) software (version 21.0; IBM, Armonk, NY, USA). Quantitative variables were presented as numerical values (percentages), medians (ranges), or medians (interquartile ranges).

## Results

**Fig. 1:**
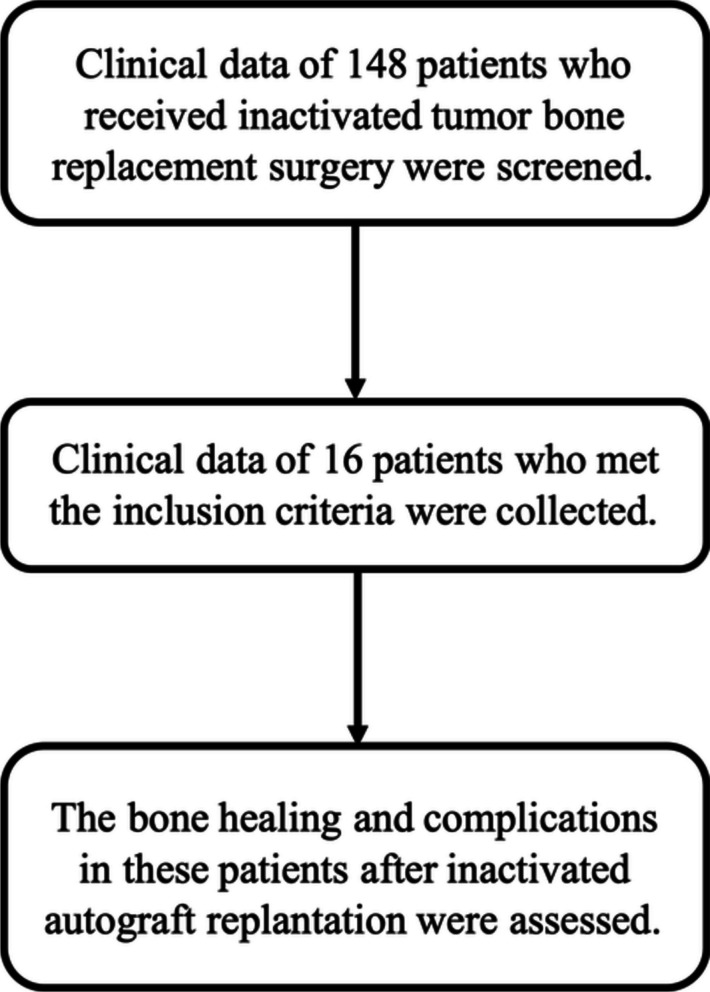
The consort diagram of the participants of this study

This study included 16 patients who met the inclusion criteria (Fig. 1 and Table 1). Among them, there were 11 males and 5 females, with an average age of 20.3 years (range, 9–54 years). Fifteen patients had osteosarcoma, and one had Ewing’s sarcoma. Nine patients had lesions in the femur and seven in the tibia. The average length of the inactivated bone was 20.2 cm (range 13.5–25.3 cm). All the patients underwent internal plate fixation.

**Table 1:** Clinical characteristics of patients who underwent knee joint activation and replantation

Patient No.	Gender	Age (year)	Histology	Primary site	Length of autograft (cm)	Inactivation method	Survival at data deadline	Bone heal of diaphysis site at data deadline	Bone healing time (month)	Complications	MSTS score
*1*	*Male*	*9*	*Osteosarcoma*	*Femur*	*20*	*Frozen*	*No*	*Yes*	*14*	*Joint fracture*	*28%*
*2*	*Female*	*13*	*Osteosarcoma*	*Tibia*	*24.2*	*Frozen*	*Yes*	*Yes*	*14*	*Joint dislocation, Joint fracture*	*23%*
*3*	*Female*	*13*	*Osteosarcoma*	*Femur*	*20.5*	*Frozen*	*No*	*Yes*	*12*	*Joint dislocation, Joint fracture*	*35%*
*4*	*Male*	*22*	*Osteosarcoma*	*Tibia*	*13.5*	*Frozen*	*Yes*	*Yes*	*27*	*Joint fracture*	*25%*
*5*	*Male*	*12*	*Osteosarcoma*	*Femur*	*18*	*Frozen*	*Yes*	*Yes*	*16*	*Joint dislocation, Joint fracture, Bone resorption*	*27%*
*6*	*Male*	*10*	*Osteosarcoma*	*Femur*	*23.5*	*Irradiation*	*No*	*No*	*-*	*Joint dislocation, Joint fracture*	*30%*
*7*	*Male*	*48*	*Osteosarcoma*	*Tibia*	*19.5*	*Irradiation*	*No*	*No*	*-*		*35%*
*8*	*Male*	*17*	*Ewing sarcoma*	*Tibia*	*17.5*	*Irradiation*	*Yes*	*No*	*-*	*Infection of inactivated bone*	*23%*
*9*	*Female*	*14*	*Osteosarcoma*	*Tibia*	*11.3*	*Irradiation*	*Yes*	*Yes*	*30*	*Joint dislocation, Joint fracture*	*28%*
*10*	*Male*	*54*	*Osteosarcoma*	*Tibia*	*17.7*	*Irradiation*	*Yes*	*No*	*-*	*Infection of inactivated bone*	*22%*
*11*	*Female*	*20*	*Osteosarcoma*	*Femur*	*30*	*Irradiation*	*Yes*	*Yes*	*28*	*Joint dislocation, Joint fracture*	*33%*
*12*	*Male*	*12*	*Osteosarcoma*	*Tibia*	*19.3*	*Ethanol*	*No*	*No*	*-*	*Recurrence*	*20%*
*13*	*Female*	*14*	*Osteosarcoma*	*Femur*	*25.3*	*Ethanol*	*No*	*No*	*-*	*Joint dislocation, Joint fracture*	*28%*
*14*	*Male*	*11*	*Osteosarcoma*	*Femur*	*17.5*	*Ethanol*	*No*	*No*	*-*	*Joint dislocation, Joint fracture, Plate fracture*	*25%*
*15*	*Male*	*23*	*Osteosarcoma*	*Femur*	*23*	*Ethanol*	*No*	*No*	*-*		*27%*
*16*	*Male*	*33*	*Osteosarcoma*	*Femur*	*22.5*	*Ethanol*	*Yes*	*Yes*	*36*	*Joint dislocation, Joint fracture*	*37%*

*MSTS*, Musculoskeletal Tumor Society

**Fig. 2:**
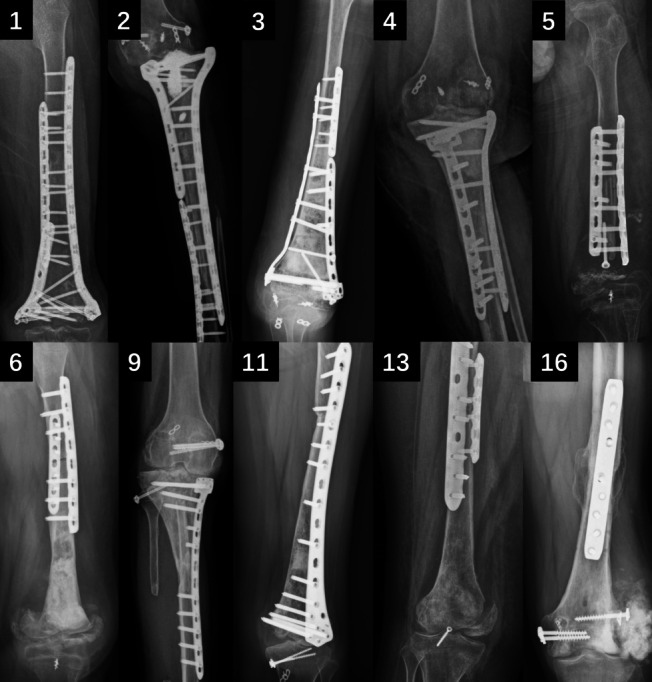
X-rays of cases of joint dislocation or fracture. Cases 1, 2, 3, 4, 5, 6, 9, 11, 13, and 16 all showed varying degrees of fractures at the joint end of the inactivated bone. Cases 2, 3, 5, 6, 9, 11, 13, and 16 are accompanied by varying degrees of joint dislocation. Except for cases 6 and 13, all of the above cases achieved bone healing at the shaft site of the inactivated bone

The average follow-up duration was 30 months (range 8–60 months). Before the data deadline of this study, eight (50%) patients were still alive, and eight (50%) died of sarcoma metastasis. Eight (50%) patients achieved bone healing at the diaphysis site of the inactivated tumor bone, with an average bone healing time of 21.9 months (range, 12–36 months). Five (31%) patients died due to metastases and did not achieve bone healing. Two (12.5%) patients did not achieve bone healing because of infection, and one (6.3%) patient underwent amputation due to tumor recurrence. Ten (62.5%) patients experienced fractures around the joint ends of the inactivated replanted bone, and eight of these ten patients were combined with joint dislocation (Fig. 2). Until the end of follow-up, the average limb function score (MSTS scale) of these 16 patients was only 28% (Table 1).

## Discussion

In this retrospective case series study, we retrospectively collected the clinical data of 16 patients with sarcoma treated with knee joint inactivation and replantation. In these patients, eight (50%) patients achieved bone healing at the diaphysis site of the inactivated tumor bone, and ten (62.5%) patients experienced fractures at the joint ends of the inactivated replanted bone, and eight of these ten patients were combined with joint dislocation.

Large bone defects resulting from musculoskeletal tumors represent tissue deficits that cannot heal spontaneously, even with adequate care and surgical stabilization [4, 5]. The purpose of surgical treatment of such large bone defects is to reconstruct the defect, avoid amputation, and provide acceptable functional results [5, 6]. The knee joint is the main load-bearing area of the human body, making it more difficult to reconstruct the surrounding large bone defects [4, 7]. Artificial prosthesis replacement is the most commonly used method for knee joint reconstruction after malignant tumor resection. Prostheses have the advantages of immediate stability, rapid recovery, and early weight bearing [8]. However, its drawbacks, including infection, mechanical loosening, mechanical wear, and fractures around the prosthesis, are evident [7, 9, 18]. The consequences of these shortcomings of knee joint prostheses are often severe [2, 7].

**Fig. 3:**
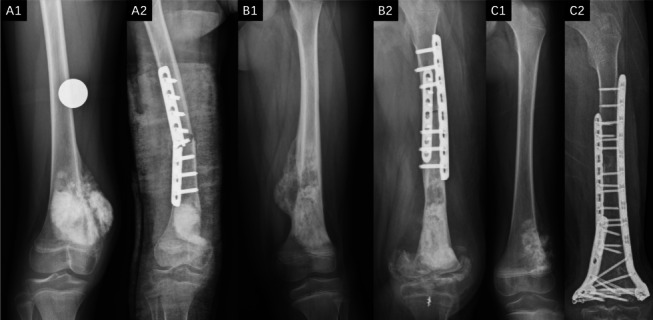
Representative cases of different internal fixation strengths in tumor bone inactivation and replantation surgery. A1 is preoperative X-rays of a patient with osteosarcoma in the right femur, and A2 shows the X-ray of this patient’s fracture 4 months after surgery. B1 is preoperative X-rays of a patient with osteosarcoma in the left femur, and B2 shows the X-ray of this patient's fracture 12 months after surgery. C1 is preoperative X-rays of a patient with osteosarcoma in the left femur, and B2 shows the X-ray of this patient’s fracture 20 months after surgery

**Fig. 4:**
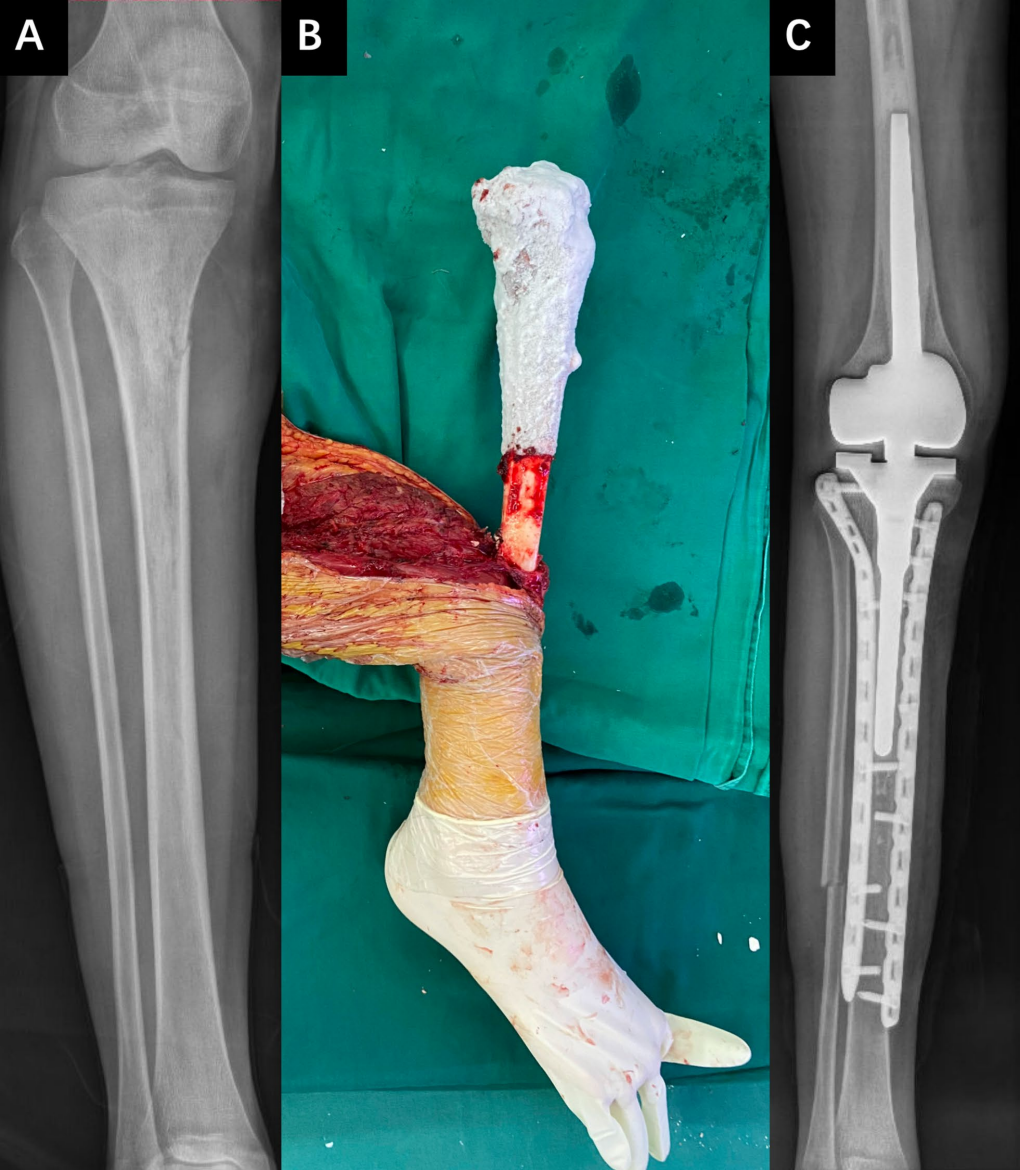
A patient with osteosarcoma in the upper right tibia underwent inactivation and replantation of the tibia, combined with artificial joint prosthesis replacement surgery

Although the application scenarios have limitations, the inactivation and replantation of tumor bone can prevent these defects in artificial prostheses. The results of this study indicate that whether it is alcohol, irradiation, or freezing inactivation, the non-joint end of the inactivated replanted bone can achieve effective bone healing, which is a feasible method. Unfortunately, regardless of the inactivation method, joint dislocations or fractures occur at almost all joint ends of the inactivated replanted bone, resulting in poor limb function and quality of life for patients after surgery. The occurrence of joint dislocation is related to insufficient blood supply to inactivated bones and ligaments. Wearing external joint braces may reduce the incidence and degree of joint dislocations. However, fractures seem inevitable. Although many studies have suggested that most inactivation methods do not reduce the mechanical strength of inactivated bones [19–21], almost all patients in this study experienced fractures after long-term follow-up (> 12 months). This indicated that the instrument strength of the inactivated bone gradually decreased before revascularization. The inevitable occurrence of fractures makes the survival of articular cartilage irrelevant. In cases where the inactivated bone is prone to fracture, the plate used to fix the inactivated bone must be long enough to cover the entire inactivated bone segment. The use of bilateral plates running through the entire inactivated bone was the most reliable internal fixation method (Fig. 3). To overcome this problem, we propose a surgical approach using tumor bone inactivation and implantation combine with an artificial prosthesis (Fig. 4). This surgical method not only effectively avoids the occurrence of joint deformities and preserves the advantages of tumor bone inactivation and replantation surgery to the maximum extent (effectively preserving the patient’s precious bone, non-immune rejection, and renewability), but also avoids the defects of artificial joint prostheses (infection, mechanical loosening, and mechanical wear). Relevant studies have demonstrated the feasibility of this surgical method [22, 23].

Although this study has shortcomings such as an insufficient number of cases, a long-time span of cases, and the nature of a single-center retrospective study, it still has important reference value. This study indicates that the inactivation and replantation of non-articular tumor bone can achieve effective bone healing and might be a feasible method. However, the incidence of joint deformities after the bone-joint inactivation and replantation surgery is high and is not recommended for use. In the future, it will be necessary to accumulate more cases and longer follow-up periods to determine the key factors influencing inactivated bone healing, as well as the efficacy of tumor bone inactivation and replantation combined with artificial prosthesis.

## Conclusions

The incidence of joint deformities after the knee-joint inactivation and replantation is extremely high (almost 100%) and is not recommended for use. Surgical methods of tumor bone inactivation and replantation combined with artificial prosthesis can be considered.

## Data Availability

The dataset supporting the conclusions of this article is available on request—please contact the corresponding author.
